# Supplementary material for: Lower FGFR2 mRNA Expression and Higher Levels of FGFR2 IIIc in HER2-Positive Breast Cancer
Source: Biology (Basel). 2024 Nov 13;13(11):920. doi: 10.3390/biology13110920 (PMC11591975; doi:10.3390/biology13110920)
Supplement: Supplementary file 1 [file biology-13-00920-s001.zip › biology-3254921-supplementary.pdf]

SUPPLEMENTARY

Supplementary Table S1. *FGFR2* transcripts

Transcript ID	Name	bp	Protein	Translation ID	Biotype	CCDS	UniProt Match
ENST00000358487.10	FGFR2-206	4624	821aa	ENSP00000351276.6	Protein coding	CCDS31298	P21802
ENST00000683211.1	FGFR2-235	6964	819aa	ENSP00000508257.1	Protein coding	CCDS81515	P21802-5
ENST00000351936.11	FGFR2-203	4761	819aa	ENSP00000309878.10	Protein coding	CCDS81515	P21802-5
ENST00000613048.4	FGFR2-224	4369	732aa	ENSP00000484154.1	Protein coding	CCDS73210	D2CGD1
ENST00000684153.1	FGFR2-240	4251	670aa	ENSP00000506937.1	Protein coding		A0A804HI76
ENST00000478859.5	FGFR2-218	3990	593aa	ENSP00000474011.1	Protein coding	CCDS81514	S4R381
ENST00000682550.1	FGFR2-230	3887	704aa	ENSP00000507633.1	Protein coding	CCDS44485	P21802-20
ENST00000369061.8	FGFR2-213	3803	709aa	ENSP00000358057.4	Protein coding	CCDS44486	P21802-23
ENST00000357555.9	FGFR2-205	3690	707aa	ENSP00000350166.5	Protein coding	CCDS53584	P21802-21
ENST00000682772.1	FGFR2-231	3638	429aa	ENSP00000506848.1	Protein coding		A0A1W2PQT9
ENST00000683035.1	FGFR2-234	3615	153aa	ENSP00000507074.1	Protein coding		A0A804HIH8
ENST00000356226.8	FGFR2-204	3547	704aa	ENSP00000348559.4	Protein coding	CCDS44485	P21802-20
ENST00000638709.2	FGFR2-227	3290	429aa	ENSP00000491912.2	Protein coding		A0A1W2PQT9
ENST00000369060.8	FGFR2-212	3244	705aa	ENSP00000358056.4	Protein coding	CCDS44487	P21802-15
ENST00000457416.6	FGFR2-215	3061	822aa	ENSP00000410294.2	Protein coding	CCDS7620	P21802-3
ENST00000369059.5	FGFR2-211	3003	707aa	ENSP00000358055.1	Protein coding		E7EVR7
ENST00000360144.7	FGFR2-208	3001	680aa	ENSP00000353262.3	Protein coding	CCDS44488	P21802-22
ENST00000369058.7	FGFR2-210	2727	768aa	ENSP00000358054.3	Protein coding		A0A5S6RJB7
ENST00000369056.5	FGFR2-209	2650	769aa	ENSP00000358052.1	Protein coding	CCDS44489	P21802-17
ENST00000336553.10	FGFR2-201	2593	669aa	ENSP00000337665.6	Protein coding		H7BXU9
ENST00000346997.6	FGFR2-202	2472	819aa	ENSP00000263451.5	Protein coding	CCDS81515	P21802-5
ENST00000359354.6	FGFR2-207	1680	254aa	ENSP00000352309.2	Protein coding		P21802-14
ENST00000429361.5	FGFR2-214	1138	371aa	ENSP00000404219.1	Protein coding		H7C265
ENST00000613324.4	FGFR2-225	579	136aa	ENSP00000481464.1	Protein coding		A0A087WY21

Transcript ID	Name	bp	Protein	Translation ID	Biotype	CCDS	UniProt Match
ENST00000611527.1	FGFR2-223	435	64aa	ENSP00000484892.1	Protein coding		A0A087X2D1
ENST00000636922.1	FGFR2-226	587	144aa	ENSP00000490905.1	Non stop decay		A0A1B0GWF4
ENST00000683250.1	FGFR2-236	5108	137aa	ENSP00000506847.1	Nonsense mediated decay		A0A804HI10
ENST00000604236.5	FGFR2-222	3501	251aa	ENSP00000474109.1	Nonsense mediated decay		S4R3B2
ENST00000682904.1	FGFR2-232	2796	No protein	-	Protein coding CDS not defined		-
ENST00000490349.5	FGFR2-219	1478	No protein	-	Protein coding CDS not defined		-
ENST00000491111.1	FGFR2-220	574	No protein	-	Protein coding CDS not defined		-
ENST00000467584.1	FGFR2-217	497	No protein	-	Protein coding CDS not defined		-
ENST00000463870.5	FGFR2-216	386	No protein	-	Protein coding CDS not defined		-
ENST00000683418.1	FGFR2-237	6317	No protein	-	Retained intron		-
ENST00000684516.1	FGFR2-241	4989	No protein	-	Retained intron		-
ENST00000683029.1	FGFR2-233	3969	No protein	-	Retained intron		-
ENST00000682296.1	FGFR2-228	3318	No protein	-	Retained intron		-
ENST00000683885.1	FGFR2-239	1838	No protein	-	Retained intron		-
ENST00000683678.1	FGFR2-238	1191	No protein	-	Retained intron		-
ENST00000682400.1	FGFR2-229	846	No protein	-	Retained intron		-
ENST00000491475.1	FGFR2-221	730	No protein	-	Retained intron		-

List of human *FGFR2* transcripts downloaded from Ensembl, showing the transcript ID, gene size (bp), size of protein, translation ID, biotype, consensus coding sequence (CCDS) and UniProt identification, to link to transcript associated protein information. The first 25 transcripts shown were protein coding; 1 sample shows that it undergoes non stop decay; 2 undergo nonsense mediated decay; 5 do not have defined coding sequences; and 8 have retained introns.

Supplementary Table S2. Multivariable linear regression analysis to model the effects of clinical attributes on *FGFR2* expression levels

		**Unadjusted**		**Mutually adjusted**	
		**Coefficient (95% CI)**	**p-value**	**Coefficient (95% CI)**	**p-value**
**Model 1 (TCGA, Nature, 2012):**					
PAM50 Subtype	Luminal A	Reference		Reference	
	Luminal B	-0.11 (-0.39 ; 0.16)	0.423	-0.09 (-0.37 ; 0.18)	0.501
	HER2-enriched	**-1.09 (-1.44 ; -0.73)**	**<0.001**	**-1.08 (-1.44 ; -0.71)**	**<0.001**
	Basal-like	0.23 (-0.08 ; 0.53)	0.143	0.20 (-0.11 ; 0.52)	0.204
Age at diagnosis		-0.00 (-0.01 ; 0.00)	0.654	-0.00 (-0.01 ; 0.01)	0.508
Nodal metastasis	Absent	Reference		Reference	
	Present	**-0.29 (-0.52 ; -0.07)**	**0.011**	-0.22 (-0.45 ; 0.01)	0.062
Survival status	Deceased	Reference		Reference	
	Alive	0.08 (-0.26 ; 0.43)	0.636	0.03 (-0.31 ; 0.37)	0.867
Stage at diagnosis	Early stage	Reference		Reference	
	Late Stage	-0.04 (-0.36 ; 0.28)	0.810	0.10 (-0.21 ; 0.41)	0.539
**Model 2 (SMC, 2018):**					
IHC Subtype	ER+	Reference		Reference	
	ER+HER2+	**-0.99 (-1.38 ; -0.60)**	**<0.001**	**-0.98 (-1.37 ; -0.59)**	**<0.001**
	HER2+	**-1.41 (-1.93 ; -0.88)**	**<0.001**	**-1.43 (-1.97 ; -0.90)**	**<0.001**
	TNBC	-0.16 (-0.52 ; 0.19)	0.364	-0.21 (-0.57 ; 0.14)	0.241
Age at diagnosis		-0.01 (-0.02 ; 0.01)	0.448	0.00 (-0.01 ; 0.02)	0.621
Stage at diagnosis	Early stage	Reference		Reference	
	Late Stage	-0.32 (-0.64 ; 0.00)	0.050	-0.29 (-0.59 ; 0.01)	0.056

ER, oestrogen receptor; HER2, human epidermal growth factor receptor 2, IHC, Immunohistochemical, TNBC, triple negative breast cancer

Model 1: FGFR2 with PAM50 using data from TCGA, Nature, 2012

Model 2: FGFR2 with IHC using data from SMC, 2018

Supplementary Table S3. Median (IQR) values of *FGFR2* expression levels from cBioPortal with intrinsic subtypes, IHC subtype; HER2 score, ER, tumour subtype

	N	Median (IQR) Log transformed
**Intrinsic subtype**	**511**	
Luminal-A	230	0.256 (-0.263; 0.699)
Luminal-B	125	0.102 (-0.613; 0.809)
HER2-enriched	58	-1.492 (-1.690; -0.097)
Basal-like	98	0.146 (-1.171; 1.059)
**IHC subtype**	**163**	
ER+	90	0.189 (-0.273; 0.756)
ER+HER2+	26	-0.626 (-1.121; -0.379)
HER2+	13	-1.134 (-1.561; -0.734)
TNBC	34	-0.107 (-0.534; 0.850)
**HER2**	**622**	
HER2: 0	61	-0.030 (-0.551; 0.526)
HER2: 1+	271	0.185 (-0.327; 0.764)
HER2: 2+	200	0.073 (-0.431; 0.540)
HER2 3+	90	-0.593 (-1.493; 0.291)
**ER**	**1050**	
ER-	238	-0.214 (-0.976; 0.541)
ER+	812	0.138 (-0.342; 0.601)
**Tumor type**	**1046**	
IDC	812	0.057 (-0.599; 0.588)
ILC	206	0.181 (-0.179; 0.623)
Mixed IDC/ILC	28	0.182 (-0.177; 0.346)

ER, oestrogen receptor; HER2, human epidermal growth factor receptor 2, TNBC, triple negative breast cancer; IDC, invasive ductal carcinoma; ILC, invasive lobular carcinoma

Supplementary Table S4. Expression of *FGFR2* exon 8 and exon 9 expression levels relative to overall *FGFR2* expression levels with respect to ER, HER2 and TNBC expression.

	N	Exon 8 Median (IQR)	Exon 9 Median (IQR)
**ER**	**1136**		
ER-	259	0.0074912 (0.003694; 0.0098429)	0.0002455 (0.000093; 0.0006832)
ER+	877	0.0089183 (0.0062117; 0.010622)	0.0002769 (0.0001346; 0.0005534)
**HER2**	**796**		
HER2-	613	0.0089153 (0.0063422; 0.0105659)	0.0002659 (0.0001387; 0.0004894)
HER2+	183	0.0082967 (0.0048052; 0.0103987)	0.0004398 (0.0001803; 0.0010876)
**TNBC**	**1023**		
ER/PR+	896	0.0088923 (0.0061799; 0.0106153)	0.000277 (0.0001352; 0.0005582)
TNBC+	127	0.0078086 (0.0054925; 0.0101662)	0.0002042 (0.0000796; 0.0004543)

ER, oestrogen receptor; HER2, human epidermal growth factor receptor 2, TNBC, triple negative breast cancer
